# Genomic Comparison of Two Family-Level Groups of the Uncultivated NAG1 Archaeal Lineage from Chemically and Geographically Disparate Hot Springs

**DOI:** 10.3389/fmicb.2017.02082

**Published:** 2017-10-31

**Authors:** Eric D. Becraft, Jeremy A. Dodsworth, Senthil K. Murugapiran, Scott C. Thomas, J. Ingemar Ohlsson, Ramunas Stepanauskas, Brian P. Hedlund, Wesley D. Swingley

**Affiliations:** ^1^Department of Biological Sciences, Northern Illinois University, DeKalb, IL, United States; ^2^Bigelow Laboratory for Ocean Sciences, East Boothbay, ME, United States; ^3^Department of Biology, California State University, San Bernardino, San Bernardino, CA, United States; ^4^School of Life Sciences, University of Nevada, Las Vegas, Las Vegas, NV, United States; ^5^MetaGénoPolis, Institut National de la Recherche Agronomique (INRA), Université Paris-Saclay, Jouy-en-Josas, France; ^6^Nevada Institute of Personalized Medicine, University of Nevada, Las Vegas, Las Vegas, NV, United States

**Keywords:** extreme microbiology, microbial ecology, uncultivated archaea, NAG1 lineage, Great Boiling Spring

## Abstract

Recent progress based on single-cell genomics and metagenomic investigations of archaea in a variety of extreme environments has led to significant advances in our understanding of the diversity, evolution, and metabolic potential of archaea, yet the vast majority of archaeal diversity remains undersampled. In this work, we coordinated single-cell genomics with metagenomics in order to construct a near-complete genome from a deeply branching uncultivated archaeal lineage sampled from Great Boiling Spring (GBS) in the U.S. Great Basin, Nevada. This taxon is distantly related (distinct families) to an archaeal genome, designated “Novel Archaeal Group 1” (NAG1), which was extracted from a metagenome recovered from an acidic iron spring in Yellowstone National Park (YNP). We compared the metabolic predictions of the NAG1 lineage to better understand how these archaea could inhabit such chemically distinct environments. Similar to the NAG1 population previously studied in YNP, the NAG1 population from GBS is predicted to utilize proteins as a primary carbon source, ferment simple carbon sources, and use oxygen as a terminal electron acceptor under oxic conditions. However, GBS NAG1 populations contained distinct genes involved in central carbon metabolism and electron transfer, including nitrite reductase, which could confer the ability to reduce nitrite under anaerobic conditions. Despite inhabiting chemically distinct environments with large variations in pH, GBS NAG1 populations shared many core genomic and metabolic features with the archaeon identified from YNP, yet were able to carve out a distinct niche at GBS.

## Introduction

Archaea are found in many environments, but can be problematic to obtain in pure culture, as many are strict anaerobes or extremophiles, or are dependent upon symbiotic relationships or co-metabolisms with other organisms (DeLong, 2003; Reysenbach et al., 2006; Wrede et al., 2012; Meyer-Dombard et al., 2013). Currently there are 19 phylum-level archaeal lineages in the NCBI taxonomy, including the phylum Euryarchaeota and members of the TACK (Thaumarchaeota, Aigarchaeota, Crenarchaeota, and Korarchaeota), DPANN (Diapherotrites, Parvarchaeota, Aenigmarchaeota, Nanoarchaeota, Nanohaloarchaea, Pacearchaeota, and Woesearchaeota), and Asgard (Thorarchaeota, Odinarchaeota, Heimdallarchaeota, and Lokiarchaeota) superphyla. This is contrasted with over eighty phyla and candidate phyla currently in the Bacteria, according to NCBI Taxonomy. Yet, recognized diversity within the archaea is expanding at a rapid pace (Castelle et al., 2015; Zaremba-Niedzwiedzka et al., 2017). The majority of cultivated archaea cluster within the phyla Euryarchaeota or Crenarchaeota in the TACK super-phylum, while metagenomics and single-cell genomics investigations have led to significant progress on the characterization of the DPANN superphylum and the recently classified Asgard group (Rinke et al., 2013; Zaremba-Niedzwiedzka et al., 2017). Nevertheless, many major taxonomic groups of archaea remain poorly represented or completely lack pure cultures and high-quality genome reconstructions (Rinke et al., 2013; Hedlund et al., 2014).

Several near-complete genomic bins representing a novel, deeply branching group called “Novel Archaeal Group 1” (NAG1) were recently described from a metagenomic study of One-hundred Spring Plains Spring, which is an acidic (pH 3.5), low oxygen, ferric iron-precipitating geothermal spring (60–78°C) in Yellowstone National Park (YNP) (Kozubal et al., 2013). NAG1 organisms were suggested to be aerobic heterotrophs capable of protein and carbohydrate catabolism as well as aerobic carbon monoxide oxidation based on the presence of two gene clusters containing Form I carbon monoxide dehydrogenases and associated maturases. The authors further suggested that NAG1 represents a novel phylum-level lineage within the Archaea and named that lineage “Geoarchaeota,” based on the deep branching position of NAG1 genomes using concatenated ribosomal protein (r-protein) trees, concatenated 16S/23S rRNA gene trees, and phylogenies of other core genome components. In contrast, other studies using concatenated gene sets showed NAG1 branching from within the phylum Crenarchaeota (Rinke et al., 2013; Guy et al., 2014). One of these reports suggested that NAG1 are deeply branching members of the order Thermoproteales (phylum Crenarchaeota) based on discordance-filtered concatenated gene trees using primarily Bayesian and maximum-likelihood approaches, as well as patterns of non-universal r-protein occurrence (Guy et al., 2014). Thus, the status of candidate phylum “Geoarchaeota” is currently unsettled, and we have chosen to use the NAG1 nomenclature in the current study.

To date, 16S rRNA gene sequences and genomes from NAG1 have mainly been recovered from iron-rich acid springs in YNP (Kozubal et al., 2013), with some notable exceptions (Meyer-Dombard et al., 2013; Colman et al., 2015). NAG1 are highly abundant in One-hundred Spring Plains Spring in Norris Geyser Basin, YNP, where they appear to represent 20–55% of the microbial community, depending on the molecular method, sampling location, and temperature (i.e., distance from the source pool) (Kozubal et al., 2013). Numerous NAG1 16S rRNA genes have also been sequenced during cultivation-independent microbial surveys of chemically similar springs in the region, further indicating that NAG1 is predominant in these acidic iron mats (Kozubal et al., 2012, 2013). A subsequent study focusing on microbial colonization and succession on glass slides incubated in One-hundred Spring Plains Spring and “Beowulf Spring” showed that NAG1 archaea colonize fresh surfaces after 30 days of incubation, specifically in middle and lower vertical sections of mature biofilms (Beam et al., 2016). These results provide invaluable information on the autecology of NAG1 archaea and are generally consistent with their proposed role in heterotrophic and microaerophilic metabolism (Kozubal et al., 2013).

Somewhat surprisingly, 16S rRNA genes (Costa et al., 2009; Cole et al., 2013) and single-amplified genomes (SAGs) affiliated with NAG1 (Rinke et al., 2013) were obtained from Great Boiling Spring (GBS), Nevada. GBS is a circumneutral pH (6.6–7.4), ∼85°C geothermal spring in northwest Nevada that has abundant populations of yet-uncultivated “microbial dark matter” (MDM) populations. Six NAG1 SAGs were previously recovered from GBS as part of the Global Encyclopedia of Bacteria and Archaea-MDM project (GEBA-MDM), a large, coordinated effort to survey genomes from MDM-rich habitats (Rinke et al., 2013). These six SAGs formed a single operational taxonomic unit (OTU) defined at the 95% average nucleotide identity (ANI) level. The individual SAG assemblies ranged from 40 to 78% in estimated genome completeness, based on the presence of conserved single copy genes, and a combined SAG assembly was estimated to be ∼93% complete. However, the NAG1 genomes were not described or interpreted in detail.

This manuscript describes (1) the first detailed study of the NAG1 population inhabiting GBS, and (2) a comparison of the ecology and evolution of two uncultivated, family-level lineages of NAG1 from chemically distinct environments. Previously sequenced and co-assembled SAGs from NAG1 and other prominent members of the GBS sediment microbial community (Rinke et al., 2013) were used as anchors to separate metagenomic reads from a sediment metagenome from GBS using nucleotide trimer frequencies and self-mapping algorithms. This approach of read binning prior to assembly has the potential advantage of reducing the possibility of artifacts due to the inclusion of DNA from an entire community during sequence assembly (Becraft et al., 2015), compared to more commonly used assembly-based binning methods (Ultsch and Morchen, 2005; McHardy et al., 2007; Strous et al., 2012). The comparison of SAGs and assembled metagenomes can also help fill the gaps in gene pathways and increase confidence in annotated metabolisms (Dodsworth et al., 2013; Becraft et al., 2015). NAG1 genomic assemblies from GBS were similar to genomes described from One-hundred Spring Plains Spring in predicted genome size, and shared many core metabolic genes despite being from chemically and geographically different hot springs, yet there were also many interesting, environment-specific metabolic differences related to central carbon metabolism and energy conservation. This work is part of a coordinated effort to identify and catalog MDM lineages and their genomic features, which improves our understanding of the diversity and evolution of life on Earth and the specific contributions of MDM to biogeochemistry.

## Materials and Methods

### Single-Cell Genomics Sampling, Sequencing, and Assembly

Samples for single-cell genomics were collected from the B site (Cole et al., 2013) within GBS located in Gerlach, NV, United States (40°39′41.16″ N, 119°21′58.5″ W) on July 22, 2009 (Rinke et al., 2013). Cells were separated from sediment by centrifugation over a Nycodenz cushion (Lindahl and Bakken, 1994), immediately frozen on dry ice, and stored at -80°C in betaine (6%, weight/volume). Cells were sorted by fluorescence-activated cell sorting (FACS), lysed, subjected to whole genome amplification, and screened by 16S rRNA gene PCR and Sanger sequencing at Bigelow Laboratory Single Cell Genomics Center (scgc.bigelow.org), as described (Rinke et al., 2013). Selected SAGs were shotgun sequenced using the Illumina HiSeq 2000 platform, filtered, and assembled using Velvet at the U.S. Department of Energy Joint Genome Institute, as described in detail previously (Rinke et al., 2013). Six SAG assemblies from GBS (Supplementary Table S1) were identified as belonging to NAG1, ranging from 0.8 to 1.15 Mbp (≥95% ANI), and raw reads from all SAGs were co-assembled yielding a 1.41 Mbp composite genome (Rinke et al., 2013).

### Metagenomics Sampling, Sequencing, and Assembly

Sediment samples for metagenomic analyses were collected from the A site in GBS on 2 December, 2008 (Cole et al., 2013). DNA was extracted from sediments using the Fast DNA Spin Kit for Soil (MP Biomedicals, Solon, OH, United States). Library preparation and sequencing of the GBS metagenome using the 454-FLX platform with Titanium chemistry (Roche, Branford, CT, United States) was performed at the Joint Genome Institute, and reads were assembled with SPAdes using kmer values of 55, 77, 99, 111, and 127 (Bankevich et al., 2012). Genome completeness and gene duplications (i.e., potential contamination) were estimated with CheckM (Parks et al., 2015) software. GBS metagenome assemblies and SAG accession numbers are reported in Supplementary Table S1.

### Read-Binning Based on Machine Learning and Prediction of Metabolic Functions

Metagenomic reads were binned by nucleotide trimer frequencies using the Multi-Layer Perceptron (MLP) machine learning package in WEKA version 3.6 (Witten et al., 2011) as described in detail in Becraft et al. (2015). The MLP was initially trained using nucleotide frequencies of clipped genomic segments of 2,000 bp from the Calescamantes SAG coassembly. In addition to the NAG1 SAG co-assembly, SAGs representing other known populations in GBS [Fervidibacteria (JGI_0000001-G10), *Thermoflexus hugenholtzii* (JGI_2140918011) (Dodsworth et al., 2014), Calescamantes (JGI_2527291514), and Aigarchaeota (JGI_2264867219)], were included to provide multiple points of reference for the algorithm (Rinke et al., 2013). Metagenomic reads were assigned to the GBS Calescamantes population if their MLP confidence score was >0.9 (a score of 1 indicates 100% confidence), assessed as the point at which false positives were minimized while maximizing true positives. The annotated genes from the NAG1 SAG co-assembly were searched against the unassembled GBS metagenomic nucleotide database using BLAST (Altschul et al., 1990), and matches with an e-value ≤ 1E-15 were also included in the assembly after removal of redundant sequences. MLP-assigned (32,699) and BLAST-identified (5,323) reads were assembled as described in Becraft et al. (2015), and assembled contigs were uploaded to RAST (Aziz et al., 2008) for gene calling and a combination of RAST and BlastKOALA (KEGG) (Kanehisa et al., 2016) were used for annotation and metabolic mapping. Functional analyses of select proteins were also predicted using CDD/SPARKLE (Marchler-Bauer et al., 2017). CRISPR regions were identified with CRISPRfinder at http://crispr.i2bc.paris-saclay.fr/Server/ (Grissa et al., 2007). Individual SAG data are located at http://microbialdarkmatter.org (Supplementary Table S1). The NAG1 metagenome assembled genome was deposited in the Integrated Microbial Genome database (IMG genome ID 2751185538)^1^.

### 16S rRNA Analysis

NAG1 SAG 16S rRNA gene sequences were queried against the GenBank NCBI-nr database using BLAST to identify the nearest sequenced relatives. 16S rRNA gene sequences within ∼85% nt identity to NAG1 sequences, as well as more distant taxa and 16S rRNA gene sequences, were aligned with SILVA (SINA package) (Quast et al., 2013) (Supplementary Figure S1). Maximum-likelihood phylogenies were generated using Mega 6.0 using the General TimeReversible (GTR) Model, with Gamma distribution with invariable sites (G+I), and 95% partial deletion with 1000x bootstrapping (Tamura et al., 2013). ANI and average amino acid identity (AAI) were calculated using the calculator at http://enve-omics.ce.gatech.edu/ani/ (Goris et al., 2007).

## Results and Discussion

### Genomic Assembly Analyses

A total of 1,548 reads from the GBS sediment metagenome obtained from BLAST and 27,462 reads from MLP classification were assembled into 250 contigs ranging from 506 to 62,608 bp (Becraft et al., 2015). The combination of small genome size and low taxonomic diversity of the NAG1 population in GBS allowed for a near-complete assembly. The metagenomic assembly was 1.4 Mbp in size out of an estimated 1.6 Mbp, representing ∼91% of the genome based on the presence of single-copy marker genes (Parks et al., 2015), from which RAST identified 1,620 predicted coding sequences (**Table 1**). The MLP metagenome assembly contained 1,595 predicted coding regions, only 60 of which were not found in the SAG co-assembly. The GBS MLP only metagenome assembly did not contain 16S or 23S rRNA genes, likely because rRNA regions have different selection pressures on their nucleotide word frequencies (Wang and Hickey, 2002). The recovery of these regions was accomplished using BLASTN with SAG 16S and 23S rRNA gene sequences as queries against the unassembled metagenome. Recovered reads were assembled, yielding full-length 16S and 23S rRNA gene sequences that were 100% identical to the SAG co-assembly. While both assemblies were high quality as suggested in Bowers et al. (2017) (>90% complete and <5% contamination), the comparison of the metagenomic assembly to the SAG co-assembly identified 137 assembly-specific proteins that filled important gaps in metabolic pathways [e.g., phosphate transport system protein PstA (JGI locus tag YNPFFACOM1_00874), arsenite oxidase (JGI locus tag YNPFFACOM1_00235), holliday junction resolvase Hjr (E.C. 3.1.22.4), and Cu^+^-exporting ATPase CopA (E.C. 3.6.3.54)]. The SAG co-assembly and MLP metagenome assembly shared 99.95% ANI, and both GBS assemblies shared ∼76% ANI (too low to accurately calculate), and 55.67% AAI, to the previously sequenced NAG1 assembly from One-Hundred Spring Plains Spring at YNP (Yarza et al., 2014) (Supplementary Table S2 and Supplementary Figure S2).

**Table 1:** NAG1 statistics for the Great Boiling Spring (GBS) single assembled genome (SAG) co-assembly, the GBS MLP metagenome assembly, and the Yellowstone National Park (YNP) metagenome assembly (Kozubal et al., 2013).

	GBS SAG co-assembly	GBS metagenome MLP assembly^1^	YNP metagenome assembly^2^
Assembly size	1,402,016	1,414,249	1,724,139
Contigs	126	91	–
Largest contig	53,594	83,401	–
N50	23	18	–
G+C content	31	28.6	32.2
Coding sequences (RAST)	1675	1595	1932
Assigned E.C. numbers (KAAS)	608	582	835
Hypothetical genes (RAST)	687 (41%)	680 (42.6%)	715 (37%)
tRNAs (RAST)	42	42	42
tRNAs (KAAS)	42	42	42
23S^3^	1	1	1
16S^3^	1	1	1
Estimated completenesss^4^	91%	93%	95%
Estimated contamination^4^	0.74%	2.21%	2.21%


*^1^Methods reported in Becraft et al. (2015). ^2^NAG1 assembly statistics are reported in Kozubal et al. (2013). ^3^16S and 23S rRNA loci were identified by BLAST in GBS MLP metagenome assembly. ^4^Estimated completeness was calculated with CheckM software.*

### 16S rRNA Gene Phylogeny

**FIGURE 1:**
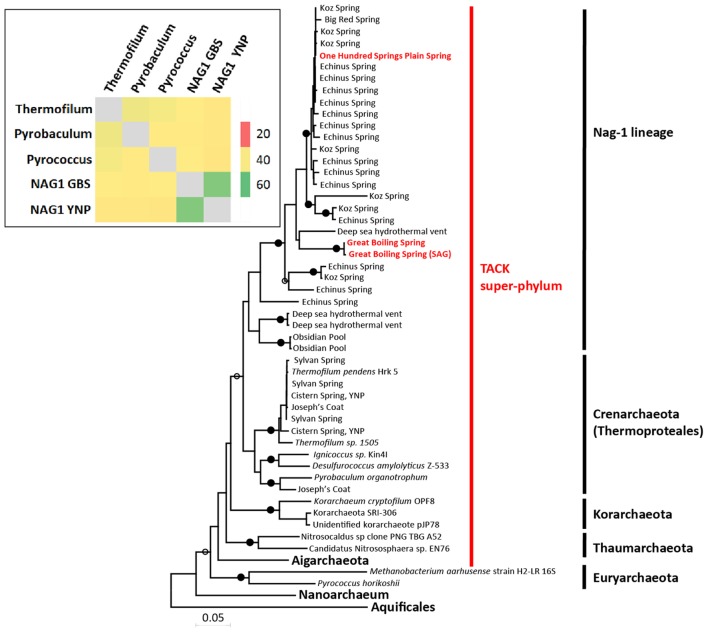
Maximum-likelihood phylogeny of partial 16S rRNA genes (1020 bp) containing sequenced members of the NAG1 phylum (>85% nucleotide identity), as well as members of the Crenarchaeota sequenced from hot springs and other well-known archaeal phyla. Hot springs locations where NAG1 sequences were isolated and sequenced are indicated next to the terminal branch in that clade. Black circles indicate bootstrap values >95%; open circles >70%. Scale bar indicates 0.05 substitutions per site. Insert (upper left); heat map of average amino acid (AAI) values among the NAG1 genomes and select genomes in the Crenarchaeota and Euryarchaeota. See Supplementary Figure S1 for GenBank IDs.

NAG1 16S rRNA gene sequences were identified previously in acidic ferric iron springs of the Lower Geyser Basin in YNP (Inskeep et al., 2013; Kozubal et al., 2013), including Koz Spring, Echinus Spring, Big Red Spring and One-Hundred Springs Plain Spring. NAG1-like sequences were also identified in Obsidian Pool in YNP (Meyer-Dombard et al., 2011), a hydrothermal vent in New Guinea (Meyer-Dombard et al., 2013) (**Figure 1**), and a Bechler region hot spring in YNP (Colman et al., 2015). NAG1-like 16S rRNA gene sequences from YNP formed two distinct clusters with ∼92% nucleotide identity; the group that included the NAG1 composite genome was comprised of sequences from Koz Spring, Echinus Spring, Big Red Spring and One-Hundred Springs Plain Spring, and the other group was comprised of sequences from Kos Spring and Echinus Spring only (Korf et al., unpublished data; Kozubal et al., 2013). The GBS SAG 16S rRNA gene sequence was 91% identical to sequences from iron springs in Norris Geyser Basin. Since this value is below the median identity separating existing bacterial families (Yarza et al., 2014), the GBS NAG1 lineage likely represents a novel family. Interestingly, NAG1 16S rRNA gene sequences at GBS were 98% identical to a partial sequence obtained from a circumneutral hot spring in the Bechler region in YNP (498 bp: GenBank ID KP091636.1), which confidently claded with the GBS NAG1 sequence, possibly representing another “species”-level population within this family lineage (Colman et al., 2015). This was the first identification of NAG1 organisms in a circumneutral hot spring. The sequence from the Bechler region hot spring was not included in **Figure 1** due to shorter length. Interestingly, the GBS NAG1 sequences were also related to an unclassified sequence obtained from a marine hydrothermal vent (87% nt identity) (Meyer-Dombard et al., 2013). The most closely related Crenarchaeota 16S rRNA gene sequences confidently identified as Thermoproteales shared 79–85% nucleotide identity with the GBS and YNP NAG1 sequences (Adam et al., 2017). NAG1 was estimated to be between 1 and 2% of the total community based on 16S rRNA sequences in Cole et al. (2013), and in this study as determined by SAG recovery, where samples were collected at ∼74°C. However, NAG1 were estimated to be >10% of the community in Costa et al. (2009), which was collected at a slightly higher temperature (79°C), indicating that NAG1 distribution and abundance could be determined by temperature similar to the acid iron springs in YNP.

### NAG1 Metabolic Potential

**FIGURE 2:**
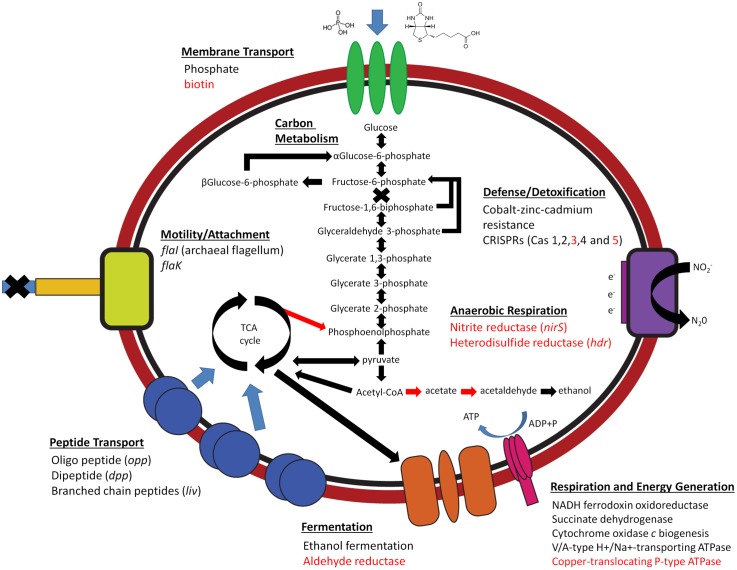
Predicted metabolisms of combined NAG1 single-amplified genome (SAG) assembly and MLP metagenomic assembly at Great Boiling Spring (GBS), Nevada. Red text and arrows indicate genes and proteins that are unique to GBS NAG1 genomes.

The GBS MLP assembly (including non-redundant BLAST hits) shared 933 predicted coding regions (out of a total of 1595; 58.5%) with the assembly from One-Hundred Springs Plain Spring (Kozubal et al., 2013). For metabolic comparisons, the annotations for the GBS SAG co-assembly and MLP assembly were pooled and treated as the same population. NAG1 genomes in both YNP and GBS did not contain genes involved with known chemolithotrophic metabolisms often found in extreme environments including iron, sulfur, hydrogen, arsenic, ammonia, or methane oxidation. In general, NAG1 populations at GBS were similar in predicted core genomic properties and metabolic function to the YNP One-Hundred Springs Plain Spring assembly (Kozubal et al., 2013) (**Figure 2** and Supplementary Figure S1). A large number of genes in the SAG co-assembly and the GBS MLP metagenome assembly were identified as hypothetical (41 and 42%, respectively), suggesting that other mechanisms of energy conservation could be present. All assemblies contained genes encoding universal ribosomal proteins and tRNA synthetases.

#### Carbon Metabolism and Respiratory Complexes

NAG1 assemblies do not appear to contain any diagnostic genes involved in autotrophic metabolisms (Berg et al., 2010). NAG1 assemblies had the potential for aerobic respiration when oxygen is available and appear to primarily utilize proteins for energy and cellular material based on membrane transport capabilities (Kozubal et al., 2013) [e.g., ABC transporters involved in dipeptide and oligopeptide transport (*opp*, *dpp*, and *liv*)] (**Figure 2**). We provide EC, KO or protein encoding gene (peg) numbers for all NAG1 metagenome assembly genes and proteins discussed in the main text; all sequences and annotations are supplied in **Supplementary Table S3**.

Similar to the YNP assembly, the NAG1 SAG co-assembly and MLP metagenome assembly from GBS contained the majority of genes for the Embden-Meyerhof-Parnas pathway (EMP) and gluconeogenesis. Of note is the absence of phosphofructokinase (PFK) (ATP-, ADP-, or diphosphate-dependent) in all assemblies. This suggests the EMP pathway is not operating in a glycolytic manner or that an unidentified enzymatic reaction fulfills the irreversible PFK-facilitated conversion of fructose 6-phosphate to fructose 1,6-phosphate. For example, the genome of *Pyrobaculum aerophilum* does not contain a PFK found in typical annotation strategies, but most likely contains an alternative kinase with shared conserved domains and similar molecular weight to PFK, capable of facilitating the PFK reaction (Fitz-Gibbon et al., 2002), though this specific protein was not identified in NAG1 at GBS. Also absent in all assemblies are glucokinases and sugar transporters, suggesting NAG1 may not utilize extracellular glucose and other sugars. However, a protein-coding sequence with slight similarity (blastp suite-2sequences; YNP = 97% cover, 5e-38 E value, 33% Identity; GBS = 63%, 2e-19, 31% identity) to a cytoplasmic ATP-dependent hexokinase from *Sulfolobus tokodaii* (Ref seq. ID WP_052847035.1) was found in the YNP assembly. The *S. tokadaii* enzyme has been shown to have broad substrate specificity, including glucose and fructose (Nishimasu et al., 2006).

The genomes suggest that the EMP and gluconeogenesis may be functioning in YNP NAG1 archaea, yet the GBS NAG1 archaea may carry out only gluconeogenesis. The YNP metagenome included a putative phosphoenolpyruvate carboxylase (E.C. 4.1.1.31), which is responsible for irreversibly supplying oxaloacetate directly into the TCA cycle through carboxylation of phosphoenolpyruvate with bicarbonate_._ Similarly, the YNP assembly included a gene coding for pyruvate kinase (E.C. 2.7.1.40), which is normally responsible for the formation of pyruvate from phosphoenolpyruvate in the final step of glycolysis. In contrast, these genes were absent from the GBS assemblies, and the gene coding for the gluconeogenic enzyme phosphoenolpyruvate carboxykinase (E.C. 4.1.1.32) was present. Although a gene encoding glucose-6-phosphatase (E.C. 3.1.1.9) was not found in any of the assemblies, all assemblies did contain malate dehydrogenase (oxaloacetate-decarboxylating) (E.C. 1.1.1.37), pyruvate orthophosphate dikinase (E.C. 2.7.9.1), and a bidirectional fructose 1,6-bisphosphate aldolase/phosphatase (E.C. 3.1.3.11). The bidirectional fructose 1,6-bisphosphate aldolase/phosphatase is thought to be an ancestral gluconeogenic enzyme and facilitates the stabilization of heat-labile triosephosphates with the creation of fructose 6-phosphate (Du et al., 2011). When NAG1 bidirectional fructose 1,6-bisphosphate aldolase/phosphatase sequences were queried using BLAST against the NCBI-nr database, all assemblies showed high similarity (99% cover and 68–70% identity) to those from members of the candidate phyla *Bathyarchaeota* and *Odinarchaeota*, as well as members of the class *Thermoprotei*, which is generally consistent with the phylogenetic placement of NAG1 (Du et al., 2011; Kozubal et al., 2013; Adam et al., 2017). This suggests that these genes were likely vertically inherited and not recently acquired.

Both YNP and GBS assemblies contained genes necessary for a complete TCA cycle. All assemblies harbor a NAD-dependent malate dehydrogenase (E.C. 1.1.1.37), with the YNP harboring an additional malate:quinone oxidoreductase (E.C. 1.1.5.4). The malate:quinone oxidoreductase has a more favorable standard free energy and has been suggested to facilitate malate oxidation under conditions where oxaloacetate:malate ratios are high (Kather et al., 2000). This may reflect the potential ability of the YNP NAG1 to feed the TCA cycle with oxaloacetate from phosphenolpyruvate using phosphenolpyruvate carboxylase (E.C. 4.1.1.31). All assemblies were found to harbor a NAD-specific isocitrate dehydrogenase (E.C. 1.1.1.41), facilitating the direct conversion of isocitrate to 2-oxo-glutarate. All assemblies appear to lack a glyoxylate cycle, suggesting NAG1 archaea do not primarily utilize small carbon compounds for anabolism.

Some genes involved in the pentose phosphate pathway were present in both YNP and GBS assemblies, including ribose 5-phosphate isomerase of the non-oxidative pentose phosphate pathway, though transketolases and transaldolases were absent. All assemblies appear to lack an oxidative pentose phosphate pathway, consistent with prior observations in archaea (Soderberg and Alver, 2004; Brasen et al., 2014), but in contrast to the interpretation of Kozubal et al. [10]. Genes involved in the ribulose monophosphate pathway (3-hexulose-6-phosphate synthases and 6-phospho-3-hexuloisomerase) were absent from all assemblies, as well as enzymes involved in the Entner-Doudoroff pathway.

All assemblies contained near-complete NADH dehydro-genase and succinate dehydrogenase complexes involved in oxidative phosphorylation, and a V/A-type ATPase (H^+^/Na^+^ transporting). The NADH dehydrogenase complex was missing *nouA, nouD, nouE* and *nouF*, though these genes have been shown to be absent in functioning NADH complexes (Friedrich and Scheide, 2000). The YNP assembly contained two gene clusters predicted to code for protein complexes capable of aerobic carbon monoxide oxidation, which were hypothesized to be involved in energy conservation (King and Weber, 2007; Kozubal et al., 2013); however, these gene clusters were absent from the GBS assemblies. The absence of genes resembling those that encode Group 1 NiFe hydrogenases, which link hydrogen oxidation to quinone reduction during chemolithotrophic hydrogen oxidation (Vignais, 2008), suggests the GBS NAG1 population does not use hydrogen as an electron donor. GBS and YNP NAG1 populations contain genes encoding cytochrome *c* oxidase complex, which is utilized to reduce oxygen as a terminal electron acceptor. NAG1 populations at YNP were also annotated to contain a cytochrome *bd* ubiquinol oxidase complex, which was absent in the GBS assemblies. Cytochrome *bd* ubiquinol protein complexes are utilized in the electron transport chain by catalyzing the reduction of oxygen under oxygen-limiting conditions, as they have a higher affinity for molecular oxygen than cytochrome *c* oxidases and can better scavenge oxygen in hypoxic environments (Das et al., 2005). Oxygen levels generally increase through diffusion along the effluent flow path of geothermal springs. Sample sites at GBS and One-Hundred Springs Plain Spring containing NAG1 populations are only a short distance from the source and had dissolved oxygen concentrations of 25–50 μM (Costa et al., 2009; Cole et al., 2013), and 22 μM (Kozubal et al., 2013), respectively. While both environments are capable of supporting populations that can utilize oxygen as an electron acceptor (Costa et al., 2009), there might be more competition for oxygen at One-Hundred Springs Plain Spring or NAG1 populations may inhabit deeper sediments. In both RAST and BlastKOALA annotations, GBS NAG1 assemblies appear to have the ability to ferment pyruvate to ethanol by oxidizing pyruvate to acetyl-CoA [2-oxoglutarate/2-oxoacid ferredoxin oxidoreductase (E.C. 1.2.7.3)], and further to acetate [acetyl-CoA synthetase; both ADP-forming (E.C. 6.2.1.13) and non-ADP-forming (E.C. 6.2.1.16)], acetaldehyde [NAD+ aldehyde dehydrogenase (E.C. 1.2.1.3)], and finally to ethanol (alcohol dehydrogenase). The YNP NAG1 assembly encoded for alcohol dehydrogenase and non-ADP-forming acetyl-CoA synthetase, it did not code for ADP-forming acetyl-CoA synthetase or aldehyde dehydrogenase (NAD+). This could indicate that the absence of the higher affinity cytochrome *bd* ubiquinol oxidase complex in NAG1 at GBS promotes a complete fermentative metabolism under hypoxic conditions. The fermentation of acetyl-CoA to acetate (*acs*) uses a bi-directional enzyme also capable of reducing acetate, though no acetate uptake proteins were identified.

#### Cofactors, Vitamins Metabolism, Mineral Acquisition, and Viral Defense

All NAG1 assemblies contained SUF genes involved in sulfur assimilation [*sufB* (K09014), *sufC* (K09013), and *sufF* (E.C. 2.8.1.7)], which is the sole method for FeS assimilation in these organisms, and all assemblies contained the *fhuD* gene involved in external Fe acquisition (K02016); the YNP assembly contained all *fhu* genes (*fhuDBC*). Additionally, genes involved in phosphate uptake [*pstSCAB* (E.C. 3.A.1.7.1) and ferrous-iron efflux transport *fieF* (K07243)] were annotated in all assemblies. All NAG1 assemblies also contained enzymes involved in co-factor production including thioredoxin [*trxA* and *trxB* (K03671 and E.C. 1.8.1.9)], adenine (E.C. 3.5.4.2) and lipoic acid (K03800), and contained pathways for oxygen stress and heavy metal detoxification, including superoxide dismutase (Fe–Mn family) (E.C. 1.15.1.1), peroxiredoxins (peg 726) and arsenic detoxification mechanisms. However, while GBS assemblies contained arsenite oxidase (JGI locus tag YNPFFACOM1_00235), they were lacking arsenic reductase and arsenic ATPase transferase. Genes coding for an archaeal-type flagellum [*flal* (K07332)] and an archaeal preflagellin peptidase [*flaK* (K07991)] were present in all assemblies, suggesting these organisms may be motile. NAG1 at YNP contained homologs to Cas1, Cas2 and Cas4, as well as a Cas10 polymerase and a region of 19 direct repeats, indicating that there are multiple active viral populations infecting NAG1 in YNP (Kozubal et al., 2013). NAG1 at GBS contained CRISPR regions identified as Cas1 (K15342), Cas2 (K09951), Cas3 (K07012), Cas4 (K07464), and Cas5 (peg 1086) and associated *cmr* genes (K19076, K09127, K09000, and K19142), though only one region of 2 and 3 repeat units were identified in the GBS MLP and SAG assemblies, respectively.

#### Unique NAG1 Metabolic Properties in GBS NAG1

NAG1 at GBS were identified as possible nitrite reducers based on the presence of a respiratory nitrite reductase gene [*nirS*: nitrite reductase (NO-forming)/hydroxylamine reductase], containing a Cytochrome cd1-nitrite reductase-like heme d1 domain, suggesting a role in nitrite respiration (E.C. 1.7.2.1) (van den Berg et al., 2000). GBS hosts a variety of thermophiles that encode partial denitrification pathways (Hedlund et al., 2011; Dodsworth et al., 2014; Becraft et al., 2015), so it is possible that the GBS NAG1 population participates in a network of metabolic handoffs, which together encode a complete denitrification pathway, which has been observed at GBS (Dodsworth et al., 2011; Hedlund et al., 2011; Rinke et al., 2013). NAG1 populations at GBS contained a copper-translocating P-type ATPase (E.C. 3.6.3.4), Na^+^/H^+^ ATPase transporters (K02123), and multiple unique genes involved in potassium uptake and homeostasis (*trk* genes) (e.g., K03499), which are necessary for growth in less acidic conditions (Padan et al., 2005). Other notable genes annotated in the NAG1 assemblies at GBS include a CoB-CoM heterosulfide reductase (*hdr*) (E.C. 1.8.98.1), which catalyzes the reversible reduction of the heterodisulfide of the methanogenic thiol enzymes coenzyme M (CoM-SH) and coenzyme B (CoB-SH). Because other genes involved in methanogenesis were not found, the role of *hdr* in the NAG1 is not clear, but it may be involved in reduction of other disulfide-containing compounds.

Populations at GBS lacked numerous genes involved in protein metabolism, including those for chorismate metabolism, a precursor to tryptophan, as well as biosynthetic pathways for tyrosine and phenylalanine. Pathways for leucine, valine, lysine, and proline biosynthesis were also absent or incomplete. This suggests that NAG1 at GBS require uptake of exogenous amino acids for protein synthesis, possibly through a symbiotic relationship with other microbes (Anantharaman et al., 2016). Calescamantes populations at GBS were also missing numerous genes involved in protein biosynthesis pathways (Becraft et al., 2015), which could indicate that available proteins are sufficient in GBS. Other genes absent in the GBS assemblies compared with the One-Hundred Springs Plain Spring genomic assembly included those involved in carbon starvation (*cstA*) and Coenzyme A and vitamin B6 metabolism (Kozubal et al., 2013). GBS NAG1 populations lacked proteins involved in tetrapyrrole heme and siroheme biosynthesis, likely due to low iron concentrations at GBS (0.23 μM), as opposed to the iron-rich acid springs they inhabit at YNP. As opposed to YNP populations, GBS assemblies lacked riboflavin synthase, and GBS populations did not contain any detoxification mechanisms for mercury. Interestingly, while no biotin utilizing enzymes were identified in Kozubal et al. (2013), probable biotin uptake genes were identified in GBS populations [*ecfT* (K16785) and *ecfA1* (K16786)]. However, proteins were not identified in GBS that utilize biotin as a cofactor, indicating a possible novel biotin utilizing enzyme, and the first organism in the TACK super-phylum lineage to utilize biotin as a cofactor (Kozubal et al., 2013).

## Conclusion

NAG1 were found in higher abundance in acid ferric iron hot springs in Yellowstone, Norris Geyser Basin compared to the circumneutral GBS, ranging between 20 and 50% of the communities at YNP versus 1–10% at GBS (Costa et al., 2009; Kozubal et al., 2013), with populations more abundant at higher temperatures. NAG1 were also identified in deep sea hydrothermal vents, or “black smokers,” which usually have lower pH ranges (≤4.5) and higher levels of Fe and S (Reysenbach et al., 2006), making GBS the most alkaline system where NAG1 have been identified in notable abundance. In both GBS and Norris Geyser Basin springs, it is likely that NAG1 populations inhabit environments that range from anoxic to hypoxic, and thus have the capability to acclimate to changing conditions. NAG1 were not identified in an appreciable extent in Little Hot Creek in the Great Basin, Octopus Spring in YNP (Inskeep, 2013), or Gongxiaoshe Spring in the Tengchong Province, China (Hou et al., 2013; Becraft et al., 2015), which have some similarity in microbiology and geochemistry with GBS. However, these organisms appear to carve out a unique niche at GBS. Interestingly, many similar organisms inhabit the communities at GBS and the acidic ferric iron springs of YNP, including *Thermoflexus*, Aigarchaeota, Fervidibacteria, and/or Calescamantes (Hedlund et al., 2011; Inskeep, 2013; Kozubal et al., 2013; Becraft et al., 2015), which indicates that community composition could be a driving force in niche formation for the NAG1 lineage.

## Author Contributions

EB was the primary author and did the bulk of data analysis. WS employed EB and provided scientific guidance and lab equipment. BH provided samples, and assisted with scientific guidance. ST assisted with metabolic reconstruction. SM helped with bioinformatics. JD contributed samples and scientific guidance. RS sorted and sequenced cells and provided scientific guidance. JO assisted in data analysis.

## Conflict of Interest Statement

The authors declare that the research was conducted in the absence of any commercial or financial relationships that could be construed as a potential conflict of interest.

## Footnotes

1 https://img.jgi.doe.gov

## Supplementary Material

TABLE S3 Pairwise comparison values of average amino acid identity (AAI) between NAG1 at Great Boiling Spring (GBS), the NAG1 YNP metagenome assembly [9], and select genomes from the Crenarchaeota and Euryarchaeota (also see **Figure 1**). GBS NAG1 SAG and MLP assemblies shared 99.95% average nucleotide identity (ANI).
