# Supplementary material for: Genomic Comparison of Two Family-Level Groups of the Uncultivated NAG1 Archaeal Lineage from Chemically and Geographically Disparate Hot Springs
Source: Front Microbiol. 2017 Oct 31;8:2082. doi: 10.3389/fmicb.2017.02082 (PMC5671600; doi:10.3389/fmicb.2017.02082)
Supplement: Supplementary file 2 [file Table_1.pdf]

Supplemental Table 1. IMG IDs and Genbank accession numbers for NAG1 single assembled genomes (SAGs) and metagenomic data sets. All individual SAG data are also located at http://microbialdarkmatter.org/index.php/mdm-project/4-single-cell-data.

NAG1 SAG assemblies [1]	IMG Genome ID	Genbank Accession #
Crenarchaeota archaeon SCGC AAA471-B05	2264867001	AQYM00000000
Crenarchaeota archaeon SCGC AAA471-B23	2264867002	ASPO00000000
Crenarchaeota archaeon SCGC AAA471-L13	2264867003	AQTC00000000
Crenarchaeota archaeon SCGC AAA471-L14	2264867268	AQSU00000000
Crenarchaeota archaeon SCGC AAA471-O08	2264867269	ASMI00000000
Crenarchaeota archaeon SCGC AAA471-C03	2264867000	ASMJ00000000
**Metagenomes**		
Great Boiling Spring sediment metagenome[2]	2053563014	N/A

[1] SAGs and co-assembly from Rinke et al. 2013.
[2] Obtained from samples collected at site A described in Cole et al. 2013 on 2 December 2008 (80 °C).
