# Supplementary material for: Genomic Comparison of Two Family-Level Groups of the Uncultivated NAG1 Archaeal Lineage from Chemically and Geographically Disparate Hot Springs
Source: Front Microbiol. 2017 Oct 31;8:2082. doi: 10.3389/fmicb.2017.02082 (PMC5671600; doi:10.3389/fmicb.2017.02082)
Supplement: Supplementary file 3 [file Table_2.pdf]

Supplemental Table 2. Pairwise comparison values of average amino acid identity (AAI) between NAG1 at Great Boiling Spring (GBS), the NAG1 YNP metagenome assembly [9], and select genomes from the Crenarchaeota and Euryarchaeota (also see Figure 1). GBS NAG1 SAG and MLP assemblies shared 99.95% average nucleotide identity (ANI).

	Thermophilum	Pyrobaculum	Pyrococcus	Geoarchaea GBS	Geoarchaea YNP
**Thermophilum**		41.29	40.29	38.94	38.33
**Pyrobaculum**	41.29		38.79	38.72	38.33
**Pyrococcus**	40.29	38.79		38.95	38.1
**Geoarchaea GBS**	38.94	38.72	38.95		55.64
**Geoarchaea YNP**	38.33	38.33	38.1	55.64	
