# Supplementary material for: Genomic Comparison of Two Family-Level Groups of the Uncultivated NAG1 Archaeal Lineage from Chemically and Geographically Disparate Hot Springs
Source: Front Microbiol. 2017 Oct 31;8:2082. doi: 10.3389/fmicb.2017.02082 (PMC5671600; doi:10.3389/fmicb.2017.02082)
Supplement: Supplementary file 4 [file Presentation_1.pdf]

Supplementary Information for the article entitled 'Genomic Comparison of two Family-Level Groups of the uncultivated NAG1 Archaeal Lineage from Chemically and Geographically Disparate Hot Springs'.

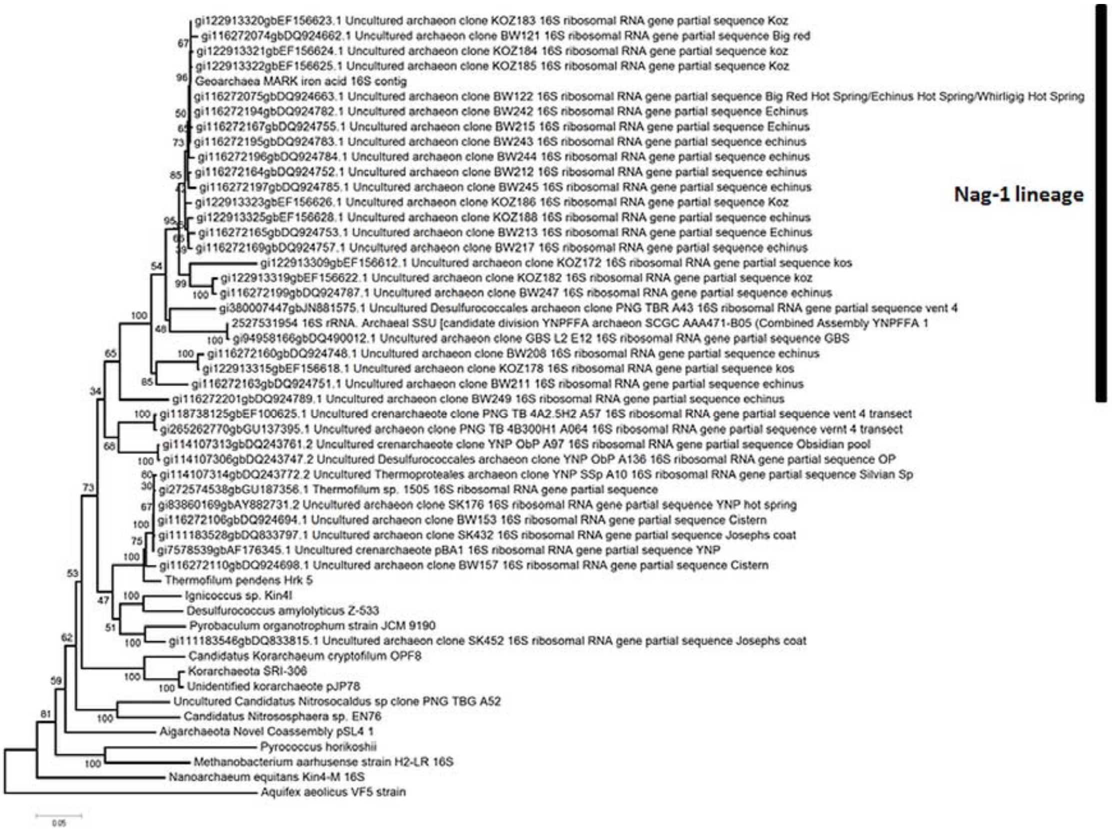

Supplemental Figure 1. Unrooted 16S rRNA gene tree used in Figure 1 with Genbank accession numbers. Black bar corresponds to the estimated NAG1 lineage.

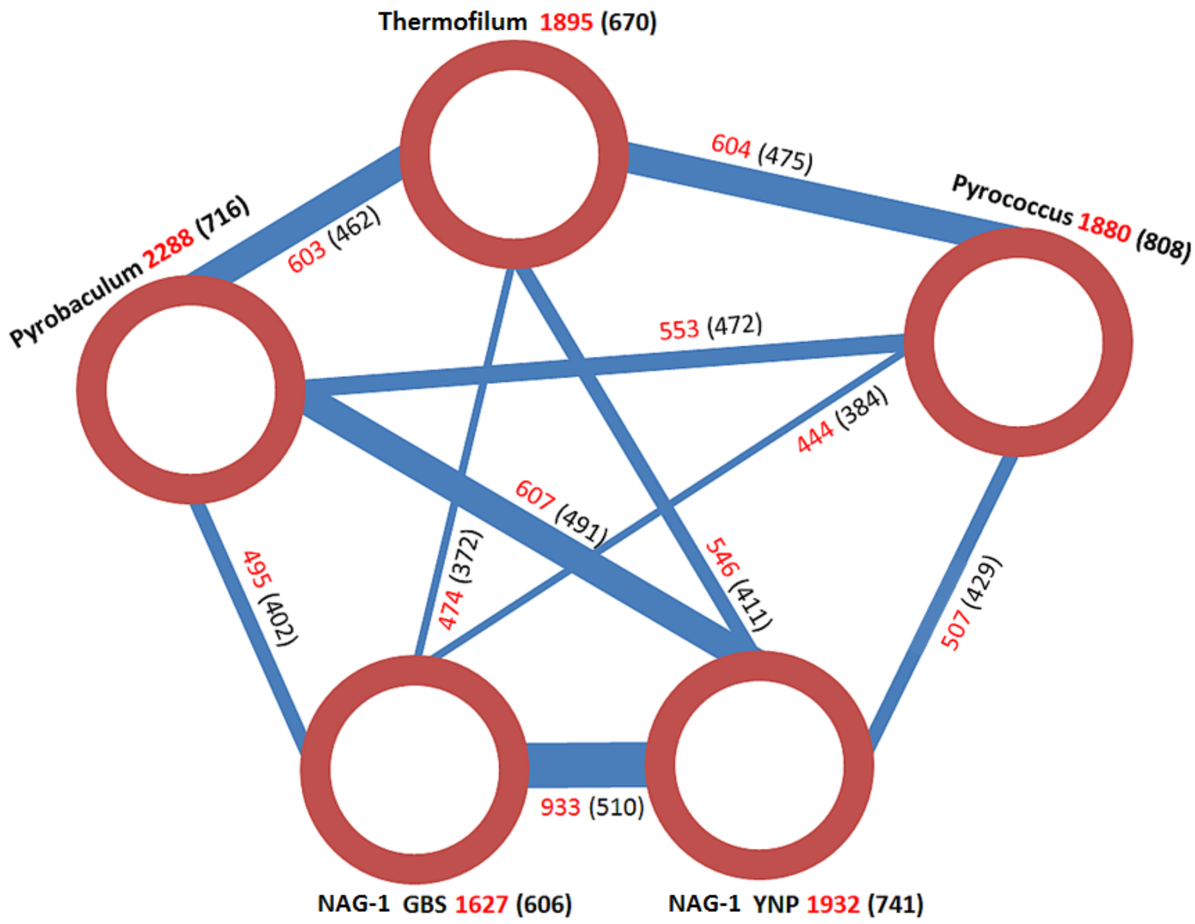

Supplemental Figure 2. Comparison of KAAS (KEGG) identified KO numbers (black) and reciprocal BLASTP hits (red) of select genomes in the Crenarchaeota and Euryarchaeota phyla to those of NAG1 metagenome assemblies from Yellowstone and Great Boiling Spring.
